# Do different multi-segment foot models detect the same changes in kinematics when wearing foot orthoses?

**DOI:** 10.1186/s13047-022-00574-z

**Published:** 2022-09-08

**Authors:** Tomas Klein, Graham J. Chapman, Ondrej Lastovicka, Miroslav Janura, Jim Richards

**Affiliations:** 1 Faculty of Physical Culture, Palacký University Olomouc, třída Míru 117, Olomouc, 77147 Czech Republic; 2 Allied Health Research Unit, University of Central Lancashire, Preston, PR1 2HE UK

**Keywords:** Multi-segment foot models, Oxford Foot Model, Foot orthoses, Biomechanical response

## Abstract

**Background:**

Different multi-segment foot models have been used to explore the effect of foot orthoses. Previous studies have compared the kinematic output of different multi-segment foot models, however, no study has explored if different multi-segment foot models detect similar kinematic changes when wearing a foot orthoses. The aim of this study was to compare the ability of two different multi-segment foot models to detect kinematic changes at the hindfoot and forefoot during the single and double support phases of gait when wearing a foot orthosis.

**Methods:**

Foot kinematics were collected during walking from a sample of 32 individuals with and without a foot orthosis with a medial heel bar using an eight-camera motion capture system. The Oxford Foot Model (OFM) and a multi-segment foot model using the Calibrated Anatomical System Technique (CAST) were applied simultaneously. Vector field statistical analysis was used to explore the kinematic effects of a medial heel bar using the two models, and the ability of the models to detect any changes in kinematics was compared.

**Results:**

For the hindfoot, both models showed very good agreement of the effect of the foot orthosis across all three anatomical planes during the single and double support phases. However, for the forefoot, the level of agreement between the models varied with both models showing good agreement of the effect in the coronal plane but poorer agreement in the transverse and sagittal planes.

**Conclusions:**

This study showed that while consistency exists across both models for the hindfoot and forefoot in the coronal plane, the forefoot in the transverse and sagittal planes showed inconsistent responses to the foot orthoses. This should be considered when interpreting the efficacy of different interventions which aim to change foot biomechanics.

## Background

Multi-segment foot kinematic modelling has been gaining popularity in foot biomechanics research with approximately forty different multi-segment foot models (MFMs) being reported in the scientific literature [1]. The evidence shows that MFMs are able to distinguish between pathological from control populations, distinguish between various foot types, assess the outcome of surgery, and provide valuable information in foot orthotics research [2–5].

A widely used MFM is the Oxford Foot Model (OFM) [6, 7], which has been used in children [7, 8] and adult [6, 9, 10] populations, and has been shown to be able to detect differences between pathological and control populations including; people with flat feet [11] and patellofemoral pain [12]. In the majority of cases the OFM has been used whilst walking barefoot and to a lesser extent when investigating the effect of foot orthoses in shod conditions. Previous research has demonstrated that the OFM’s within-session and between-day reliability for in-shoe overground [13] and treadmill [14] walking is comparable to data during barefoot walking. Under shod conditions, the OFM has shown kinematic differences between individuals with midfoot pain compared to healthy individuals [15] and the kinematic effect of different foot orthoses in participants with midfoot osteoarthritis [16], as well as differences in knee adduction moments when used in conjunction with the Plug-in Gait marker set [17].

An alternative concept is a multi-segment foot model (MFM) based on the Calibrated Anatomical System Technique (CAST) to model the different foot segments in six degrees of freedom (6DOF) [18]. The CAST MFM has been previously used with footwear to determine ankle and metatarsophalangeal stiffness during walking and jogging [19], and to explore the effect of foot orthoses on foot kinematics [5].

It has been shown that different foot models produce different outputs due to different marker sets and anatomical axes definitions in both static and dynamic conditions [20, 21]. Therefore, differences can be also anticipated for the OFM and CAST MFM, however whether these actually exist and how these may affect the interpretation of foot kinematics remains uncertain. Previous research has focused on the comparison of the kinematic outputs of various MFMs [20–22], however the translation of this information to explore the effect of interventions such as the effect of foot orthoses remains unexplored. The examination of different MFMs’ ability to detect kinematic changes would provide a useful comparison of the effect of foot orthoses, where even subtle differences in kinematics can potentially have an impact on interpretation of the data such as identification of biomechanical responders and non-responders. Therefore, the aim of this study was to compare the ability of the CAST MFM and the OFM to detect changes in hindfoot and forefoot kinematics during the stance phase of gait when wearing a foot orthosis.

## Methods

### Participants

A convenience sample of healthy individuals with no congenital, or acquired pathology of the nervous or musculoskeletal systems, no deformities or serious injuries of the pelvis or lower limbs and feet, no self-reported lower limb/foot pain were included in the study. This study was approved by the Ethical Committee of the Faculty of Physical Culture, Palacký University in Olomouc, Czech Republic (reference number 3/2018) and all participants provided written informed consent prior to data collection.

### Procedures

**Fig. 1:**
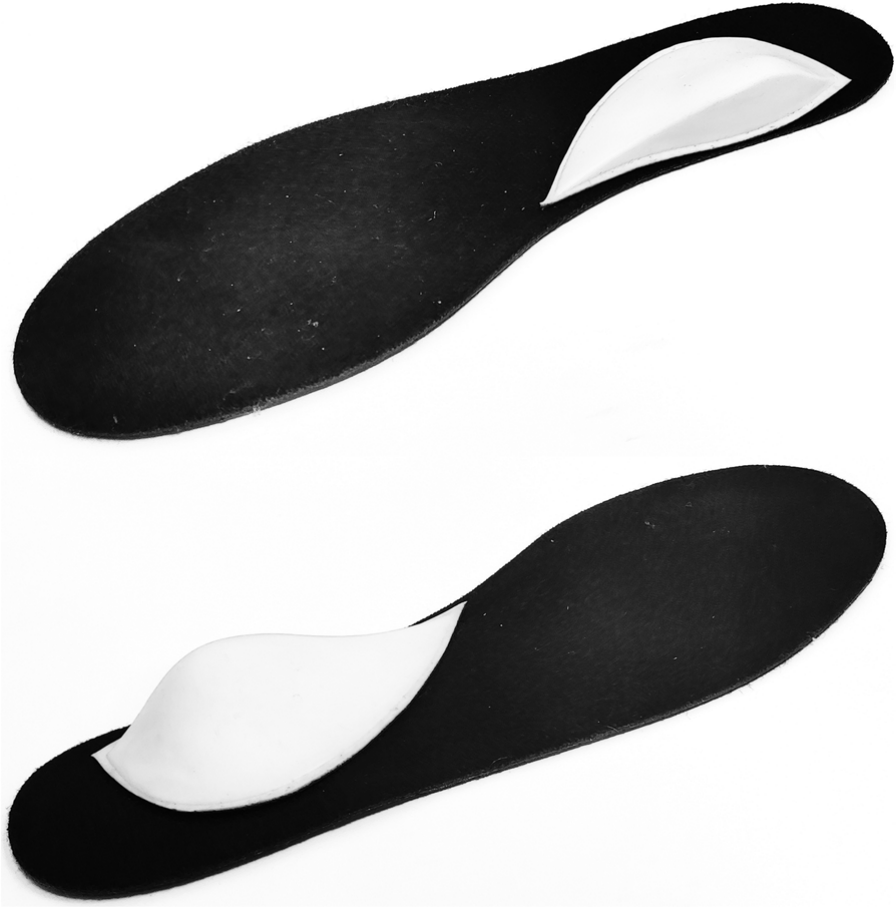
Diagram illustrating medial (top) and lateral (bottom) aspects of the medial heel bar

Participants walked at a self-selected walking speed along a 15 m walkway whilst wearing a correctly sized pair of ProTouch Drop Shot trainers (IIC-INTERSPORT, Bern, Switzerland) under two conditions; no orthosis and a pair of foot orthoses with a medial heel bar, positioned under the sustentaculum tali which aimed to minimise foot eversion during the stance phase (see Fig. 1). The OFM and CAST foot kinematics were captured simultaneously at 200 Hz using an eight camera Vicon Vantage V5 (Oxford Metrics, UK) motion capture system.

**Fig. 2:**
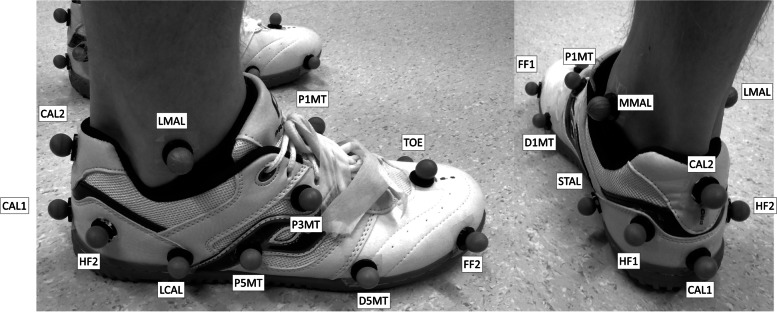
Diagram illustrating the lateral (left) and posterior-medial marker positions of the Calibrated Anatomical System Technique (CAST) multi-segment foot model and the Oxford Foot Model (OFM) applied simultaneously. CAL1 and CAL2 represents the inferior and superior posterior aspect of calcaneus; HF1 and HF2 represents the medial and lateral hindfoot; STAL represents the sustentaculum tali; LCAL represents the lateral aspect of the calcaneus (at the same distance from the most posterior point as STAL); MMAL and LMAL represents the medial and lateral malleoli; P1MT, P5MT, and P3MT represents the base of the 1^st^ and 5^th^ metatarsals, and between the base of the 3^rd^ and the 4^th^ metatarsals, respectively; D1MT and D5MT represents the medial first and lateral fifth metatarsal heads; TOE represents the mid-point of the distal heads of the 2^nd^ and 3^rd^ metatarsals; FF1 and FF2 were not used for the analysis

The CAST lower limb model was used to measure lower limb kinematics [18]. Anatomical markers were placed on the anterior and posterior superior iliac spines, medial and lateral femoral epicondyles, and medial and lateral malleoli. The shank segment was defined proximally by the knee joint center, estimated from the medial and lateral femoral epicondyle markers, and distally by markers placed on the medial and lateral malleoli. Clusters of non-collinear markers were placed on the lower leg (i.e. shank) and the thigh. Marker placement for both foot models is displayed in Fig. 2. Retroreflective markers for the OFM were positioned according to Stebbins et al. [7] with a following modifications. Instead of a marker wand positioned on the posterior aspect of the calcaneus, the lateral calcaneus (HF2) marker was used for hindfoot tracking, the sustentaculum tali marker was moved inferior to fit on the shoe surface, and the hallux was not tracked in this study due to not being able to place a marker on the hallux under shod conditions.

The proximal and distal CAST MFM hindfoot was defined by the medial and lateral malleoli (MMAL, LMAL) and sustentaculum tali (STAL) and lateral aspect of the calcaneus (LCAL, at the same distance from the most posterior point as STAL [7]) markers, respectively. The hindfoot was tracked using markers positioned on the medial and lateral hindfoot (HF1, HF2) in addition to the marker positioned inferior on the posterior aspect of the calcaneus (CAL1), and STAL and LCAL markers. The proximal and the distal forefoot were defined by the STAL and LCAL markers, and the first and the fifth metatarsal markers (D1MT, D5MT), respectively. The forefoot was tracked using markers positioned on the base of the 1^st^ and 5^th^ metatarsals, and between the base of the 3^rd^ and the 4^th^ metatarsal (P1MT, P5MT and P3MT, respectively). The two most anteriorly placed markers (FF1, FF2) were not used by either model in this analysis (Table 1).

**Table 1:** Definition of the CAST MFM and the OFM hindfoot and forefoot segments

**CAST MFM**							
	**Proximal end**		**Distal end**		**A/P axis**	**M/L axis**	**Tracking markers**
	Lateral	Medial	Lateral	Medial			
**Hindfoot**	LMAL	MMAL	LCAL	STAL	Y	X	STAL, LCAL, HF1, HF2, CAL1
**Forefoot**	LCAL	STAL	D5MT	D1MT	Y	X	P1MT, P3MT, P5MT
**OFM**							
	**Plane 1**		**A/P axis**		**M/L axis**		**Tracking markers**
**Hindfoot**	CAL1, CAL2, static midpoint between LCAL and STAL		From CAL1 in plane 1, parallel to the floor (Y)		Perpendicular to the plane 1 (X)		STAL, LCAL, CAL1, HF2
**Forefoot**	D1MT, D5MT, P5MT		Line from midpoint between P1MT and P5MT to TOE projected onto plane 1 (Y)		On plane 1, perpendicular to A/P axis (X)		P1MT, P5MT, D5MT, TOE

*CAST* Calibrated Anatomical System Technique, *MFM* Multi-segment foot model, *OFM* Oxford Foot Model, *A/P* Anterioposterior, *M/L* Mediolateral, Marker position: *CAL1 *and *CAL2* Inferior and superior posterior aspect of calcaneus, respectively, *HF1 *and* HF2* Medial and lateral hindfoot, respectively, *STAL* Sustentaculum tali, *LCAL* Lateral aspect of the calcaneus, *MMAL* and *LMAL* Medial and lateral malleoli, respectively, *P1MT, P5MT, *and* P3MT* Base of the 1st and 5th metatarsals, and between the base of the 3rd and the 4th metatarsals, respectively, *D1MT* and *D5MT* Medial first and lateral fifth metatarsal heads, respectively, *TOE* Mid-point of the distal heads of the 2nd and 3rd metatarsals

A single static trial with participant standing with a comfortable relaxed posture was captured and used to calculate local coordinate systems of the segments. Anatomical markers were removed after the static trial and all tracking markers remained in place during both conditions. For each participant, twenty-five trials were collected, of which seventeen trials had complete marker trajectories for all markers, and were used for the analysis. The two orthotic conditions were randomized by participants picking different coloured balls from a bag which represented the with and without orthotic conditions. A five-minute familiarization and wash-out period was used between each condition.

### Data analysis

Kinematic data were processed in Vicon Nexus 2.8 and exported to C3D format. Heel strike and toe-off were identified manually based on linear acceleration, velocity and visual inspection of the heel and toe marker trajectories. The kinematic data were filtered with a fourth-order low-pass Butterworth filter with a 6 Hz cut-off frequency. The CAST lower limb model and both the CAST MFM and the OFM were applied in Visual 3D (C-motion, USA) using segment optimization pose estimation. For the shank, hindfoot and forefoot segment coordinate systems of both foot models, the motion about a medio-lateral (X), an antero-posterior (Y) and a vertical (Z) axis was plantar/dorsiflexion, inversion/eversion and abduction/adduction, respectively. The X–Y-Z Cardan rotation sequence equivalent to the segment coordinate system was used to calculate joint kinematics [23], and data were normalized to 100% of the stance phase.

**Fig. 3:**
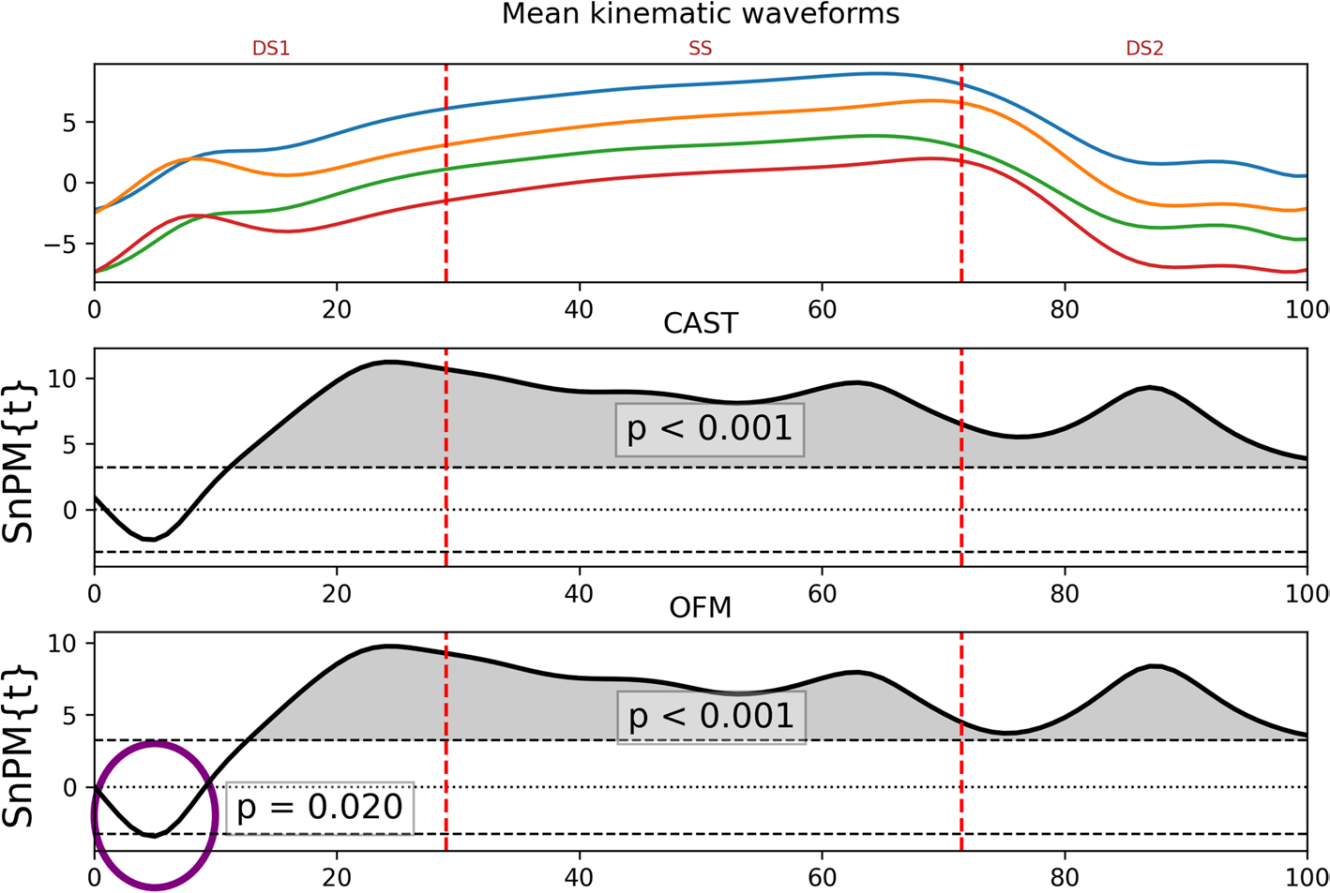
An example of statistical non-parametric mapping (SnPM) analysis conducted for both the Calibrated Anatomical System Technique (CAST) multi-segment foot model and the Oxford Foot Model (OFM) for the hindfoot coronal plane during the first and second double support sub-phases (DS1 and DS2) and single support sub-phase (SS). The upper panel shows mean kinematic waveforms for the CAST and OFM under no orthoses (blue and green, respectively) and under the medial heel bar conditions (orange and red, respectively).The lower two panels show the statistical output of the SnPM analysis with grey shaded area illustrating the suprathreshold clusters (*p* < 0.05). The purple circle highlights the suprathreshold cluster not included in the analysis

Statistical analysis was carried out using spm1d package version 0.4.3 (http://www.spm1d.org/) in Python version 3.8. The D’Agostino-Pearson K2 test was used to assess the time series data normality. Data were not normally distributed; therefore, the non-parametric version of vector field analysis, statistical non-parametric mapping (SnPM) was used [24]. SnPM paired t-tests (*p* < 0.05), with the number of iterations set to 10,000, were used to explore the effect of the medial heel bar over the stance phase for each foot model for each participant in all three anatomical planes using observations from both feet. The hindfoot relative to shank and forefoot relative to hindfoot segments in both feet were compared between models. The segments for each foot model had comparable markers and thus hypothesised that outputs would be similar, despite having subtly different anatomical axes definitions. The stance phase was split into the first double support (DS1), single support (SS) and the second double support (DS2) phase, identified from the gait events, and statistically significant kinematic effects of the medial heel bar, represented by the suprathreshold clusters in the SnPM analysis, were compared between the two foot models for each sub-phase separately. For each sub-phase of the gait cycle, possible kinematic outcomes between foot models were; the same kinematic effect (blue), no effect for both models (turquoise), a unique effect of either the CAST MFM (green) or OFM (orange), or an opposite effect of the two foot models (red). The visual inspection of the SnPM analysis outputs showed considerable number of small, arguably clinically irrelevant suprathreshold clusters (waveform areas showing statistically significant differences). The authors could not find any data to identify minimal clinically relevant suprathreshold cluster size specific to this type of analysis, therefore, based on the visual inspection, only differences that were significant for more than 5% of the stance phase were considered meaningful in the different sub-phases and were included in the analysis (Fig. 3).

## Results

**Fig. 4:**
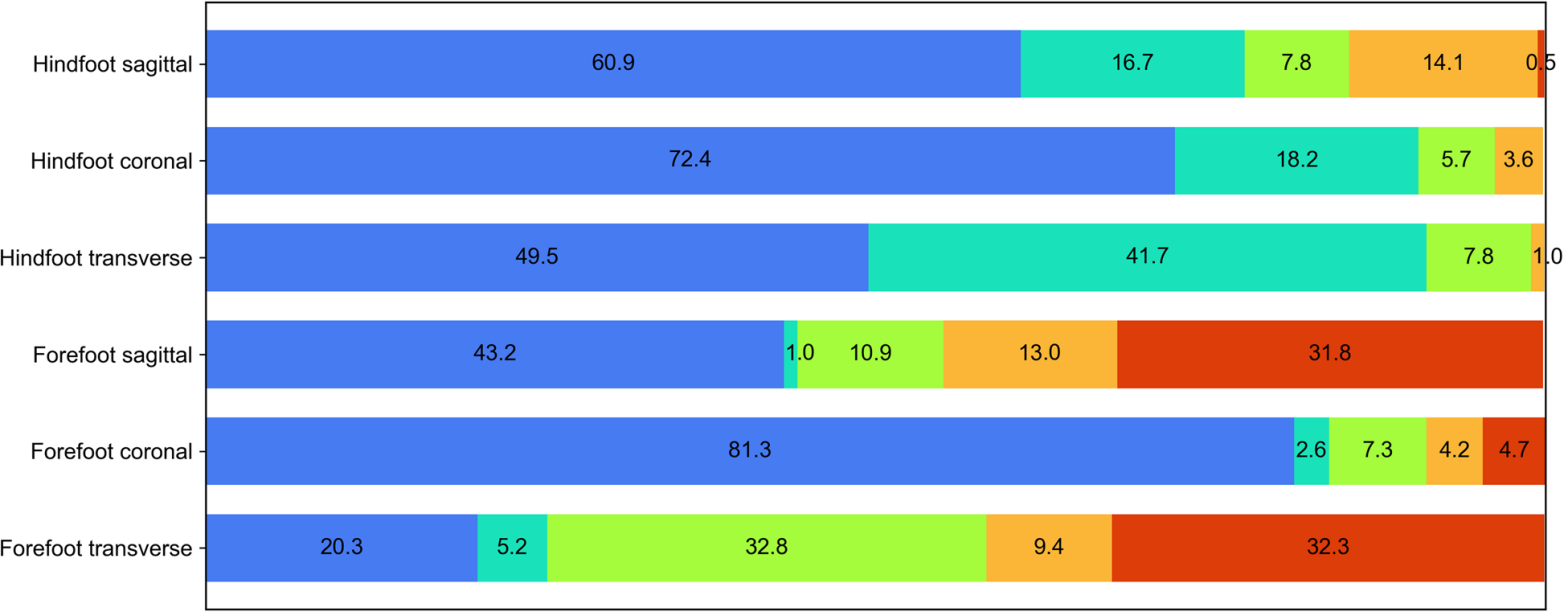
The comparison of the Calibrated Anatomical System Technique (CAST) multi-segment foot model and the Oxford Foot Model (OFM) ability to detect the effect of the medial heel bar in hindfoot and forefoot in sagittal, coronal and transverse plane. Respective colours represent the percentage of the ability to detect the same kinematic change (blue), no kinematic change (turquoise), the unique kinematic change for CAST (green) and OFM (orange) foot models and the opposite kinematic change (red)

Thirty-two participants (16 females and 16 males), who were all right side dominant, and had a mean age of 22.9 ± 3.5 years, body weight of 67.9 ± 10.4 kg and height of 173.7 ± 10.3 cm, were recruited. Both limbs showed an analogous kinematic trend under orthotic conditions, therefore results from both feet were averaged. The ability of the CAST MFM and the OFM to detect the effect of the medial heel bar in the hindfoot and forefoot in the sagittal, coronal and transverse plane are presented in Fig. 4. Results including sub-phases are presented in Table 2.

**Table 2:** Comparison of CAST MFM and OFM ability to detect the effect of medial heel bar

	DS1			SS			DS2			**Total**
	Sagittal	Coronal	Transverse	Sagittal	Coronal	Transverse	Sagittal	Coronal	Transverse	
**Hindfoot (%)**										
CAST MFM	10.9	4.7	10.9	10.9	7.8	6.3	1.6	4.7	6.3	**7.1**
OFM	14.1	4.7	0.0	14.1	0.0	3.1	14.1	6.3	0.0	**6.3**
No kinematic change	20.3	10.9	32.8	14.1	4.7	39.1	15.6	39.1	53.1	**25.5**
Opposite effect	0.0	0.0	0.0	0.0	0.0	0.0	1.6	0.0	0.0	**0.2**
Same kinematic change	54.7	79.7	56.3	60.9	87.5	51.6	67.2	50.0	40.6	**60.9**
**Forefoot (%)**										
CAST	14.1	10.9	17.2	10.9	3.1	29.7	7.8	7.8	51.6	**17.0**
OFM	14.1	1.6	9.4	17.2	7.8	10.9	7.8	3.1	7.8	**8.9**
No kinematic change	1.6	1.6	4.7	0.0	0.0	4.7	1.6	6.3	6.3	**3.0**
Opposite effect	25.0	4.7	46.9	37.5	4.7	35.9	32.8	4.7	14.1	**22.9**
Same kinematic change	45.3	81.3	21.9	34.4	84.4	18.8	50.0	78.1	20.3	**48.3**

*DS1* First double support, *DS2* Second double support, *SS* Single support, *OFM* Oxford Foot Model, *CAST* Calibrated Anatomical System Technique, *MFM* Multi-segment foot model

### Hindfoot

At the hindfoot, there was good agreement between the foot models, with both foot models detecting the same kinematic changes to the orthotic condition in 60.9% of cases, with 25.5% of cases demonstrating no kinematic differences in both foot models. The CAST MFM was able to detect unique kinematic changes in 7.8% of the cases in the sagittal plane (10.9% in DS1, 10.9% in SS, 1.6% in DS2), 5.7% of cases in the coronal plane (4.7% in DS1, 7.8% in SS, 4.7% in DS2), and 7.8% of the cases in the transverse plane (10.9% in DS1, 6.3% in SS, 6.3% in DS2). The OFM was able to detect unique kinematic changes in 14.1% of the cases in the sagittal plane (14.1% in DS1, SS, and DS2), 3.6% of the cases in the coronal plane (4.7% in DS1, 6.3% in DS2), and 3.1% of the cases in the transverse plane SS, with an opposite effect being observed in 1.6% of the cases in the sagittal plane in DS2.

### Forefoot

At the forefoot, the same overall effect of the medial heel bar was observed in 51.3% of the cases with both models being able to detect the same kinematic change in 48.3% and no kinematic change in 3.0% of the cases. The CAST MFM was able to detect unique kinematic change in 10.9% of the cases in the sagittal plane (14.1% in DS1, 10.9% in SS, 7.8% in DS2), 7.3% of the cases in the coronal plane (10.9% in DS1, 3.1% in SS, 7.8% in DS2), and 32.3% of the cases in the transverse plane (17.2% in DS1, 29.7% in SS, 51.6% in DS2). The OFM was able to detect unique kinematic change in 13.0% of the cases in the sagittal plane (14.1% in DS1, 17.2 in SS, 7.8 in DS2), 4.2% of the cases in the coronal plane (1.6% in DS1, 7.8% in DS2, 3.1 in DS2), and 9.4% of the cases in the transverse plane (9.4% in DS1, 10.9% in DS2, 7.8 in DS2). The opposite effect was observed in 31.8% of the cases in the sagittal plane (25.0% in DS1, 37.5% in DS2, 32.8% in DS2), 4.7% of the cases in DS1, SS, and DS2 in the coronal plane, and 32.3% of the cases in the transverse plane (46.9% in DS1, 35.9 in SS, 14.1% in DS2).

## Discussion

This study examined the ability of the CAST MFM and OFM models to detect changes in the hindfoot and forefoot kinematics. It is important to emphasize that this study cannot and does not attempt to say which of the models is correct. The SnPM explored the effect of the medial heel bar on foot kinematics during stance phase in both models and the results were used to compare the ability to detect a change. To the authors’ knowledge, this is the first time SnPM has been used to identify how two multi-segment foot models detect similar or opposing kinematic changes under orthoses conditions. The CAST MFM and the OFM were chosen for the comparison because they represent two different concepts in multi-segment foot modelling and both have been previously used to investigate the effect of foot orthoses. Our findings demonstrate that there was good agreement/similar detectable kinematic changes between the two foot models in the coronal plane hindfoot and forefoot kinematics, but there were considerable differences in kinematics between foot models for the forefoot in the sagittal and transverse planes.

### Hindfoot

At the hindfoot, the overall agreement of both models across all planes over the entire stance phase was very good, potentially due to the similarities in the markers used to track the hindfoot segment, despite the subtle differences in the segment coordinate system definitions. Also the use of the same biomechanical conventions could contribute to the good agreement of the models as it was suggested to be more crucial than the design of relevant marker sets in the comparison of different gait protocols [25]. Both models were in very good agreement in detecting the reduction of hindfoot eversion, which is consistent with past research on the effect of medial heel bars on foot [26] and hindfoot [5] kinematics, and rearfoot posting [27–29]. The CAST MFM detected slightly more (~ 2% of cases) unique kinematic changes than the OFM. No opposite effect was present. The small differences between models could be explained by an extra tracking marker in the CAST MFM which may improve the precision of the measures of that segment, however the effect of the differences in the segment coordinate system definitions cannot be ruled out.

In the transverse plane, both models were in very good agreement in detecting increased hindfoot abduction, which was reported to be the effect of medial heel bar on hindfoot [5] and foot [26], across all the phases. The CAST MFM was able to detect a unique change in more cases (approximately ~ 8%) across all stance sub-phases, while the OFM was able to detect a unique kinematic change (~ 3% of cases) and only in SS phase. In the sagittal plane, there was reasonable overall agreement in the ability of both models to detect increased dorsiflexion (~ 77%), which was previously identified as the effect of a medial heel bar on the hindfoot [5] and the foot [26], across all phases, however the OFM detected a unique kinematic change in approximately twice as many number of cases (~ 14%, see Table 2) across all the phases than the CAST MFM. Hindfoot results imply a minor shift between hindfoot local coordinate system axes of both models leading to a different distribution of the three dimensional joint rotations across the anatomical planes, which could potentially explain the subtle differences in the sensitivity of the models to be able to detect kinematic changes in a given plane across all phases.

### Forefoot

At the forefoot there was a marked difference in overall agreement of both foot models across all planes over the entire stance phase (approximately 50% of cases), whilst there was a marked increase (~ 23% of all cases) in opposing kinematic change between the MFM. However, this measurement of response due to the foot orthosis differed significantly across anatomical planes. The highest level of agreement of both models was observed in the coronal plane, where previous findings demonstrated the medial heel bar increased inversion of the forefoot during the entire stance phase [5]. On the contrary, the level of agreement between the CAST MFM and the OFM was rather poor in the sagittal and transverse planes (~ 45% and ~ 25% of cases, respectively) with the opposite effect detected in approximately a third of the cases in both planes. These differences could be explained by the different number and position of tracking markers with the OFM using an extra tracking marker. While the three CAST MFM tracking markers are positioned above the base of the metatarsals, two out of the four OFM tracking markers are positioned in a more anterior position (mid-point between 2^nd^ and 3^rd^ metatarsal heads and 5^th^ metatarsal head), which could possibly explain the difference in detected kinematic change as the OFM is partially tracking a more distal part of the foot. In the transverse plane, the median medial heel bar effect on the peak abduction of the forefoot was reported to be less than 0.3º degrees [5], which together with different tracking marker placements could contribute to the high percentage of disagreement between the two models.

In foot orthoses research it is common practice to report the material, density, shape and inclination of foot orthoses’ elements [27, 28] in order to explore a particular prescription and its variations [29]. Knowing the features of various MFMs is no different. The medial heel bar used in this study has been shown to decrease eversion of the hindfoot in healthy adults, which may benefit patients with abnormal hindfoot pronation [5]. Both the CAST MFM and the OFM showed very good agreement in the ability to detect kinematic changes as a result of the medial heel bar in the coronal plane (90.6% in the hindfoot, 83.6% in the forefoot), which implies both models could be used interchangeably. However, recent work has shown that the effect of foot orthoses [30] and specifically designed shoes [31] is not necessarily universal and responders and non-responders can be identified from biomechanical data. In the coronal plane of the hindfoot, the CAST MFM and the OFM detected unique kinematic changes in 5.7% and 3.6% of the cases, respectively, which shows a level of disagreement between the models in the identification of possible biomechanical responders and non-responders in 9.3% of the cases. The level of disagreement in the identification of possible biomechanical responders and non-responders in the coronal plane of the forefoot was 11.5% of cases with the CAST MFM and the OFM being able to detect a unique kinematic change in 7.3% and 4.2% of cases, respectively. This level of difference could potentially have an impact on the identification of biomechanical responders and non-responders to the hindfoot medial posting intervention. This is an important area for future work which is required to explore the ability of two models to identify responders and to determine the most appropriate model, if any. As there are different versions of the OFM producing slightly different kinematic outputs [20], the level of disagreement may change depending on the version used.

This study had some limitations. All the participants were healthy individuals therefore the magnitude of the effect and potentially the ability of both models to detect kinematic change may differ for people requiring orthotic interventions. Previous comparisons of the OFM and Rizzoli Foot Model have shown differences present in kinematic outputs which also depended on gait type [20]. Due to its position, the motion of the markers placed on the midpoint between the 2^nd^ and 3^rd^ metatarsal heads and on the 5^th^ metatarsal head could be susceptible to the deformation of the shoe, especially during the heel rise, which could have an effect on the OFM forefoot kinematics.

## Conclusion

This study examined the ability of the CAST MFM and the OFM to detect changes in the hindfoot to tibia and forefoot to hindfoot kinematics during the stance phase of gait when using a foot orthosis. While in the hindfoot the two models detected the same effect of the medial heel bar, in the forefoot this varied greatly. In the transverse and sagittal plane forefoot kinematics the agreement between the models was poor, however in the coronal plane, where the main clinical effect of the medial heel bar would be expected, both models were in good agreement for both the hindfoot and the forefoot. In both hindfoot and forefoot, both models were able to detect unique kinematic effects of the medial heel bar, which could have an impact on identifying potential biomechanical responders and non-responders. At this time this study cannot determine which of the two models offers the best option but seeks to demonstrate that different models can yield different measurement effects when considering an intervention.

## Data Availability

The datasets used and/or analysed during the current study are available from the corresponding author on reasonable request.

## Abbreviations

MFMs: Multi-segment foot models
MFM: Multi-segment foot model
OFM: Oxford Foot Model
CAST: Calibrated anatomical system technique
6DOF: Six degrees of freedom
SnPM: Statistical non-parametric mapping
DS1: First double support
SS: Single support
DS2: Second double support
CAL1, CAL2: Markers placed on posterior aspect of calcaneus, distal and proximal ends of the midline in the sagittal plane, respectively
HF1, HF2: Markers placed on medial and lateral hindfoot, respectively
STAL: Marker placed on sustentaculum tali
LCAL: Marker placed on lateral aspect of the calcaneus (at the same distance from the most posterior point as STAL)
MMAL, LMAL: Markers placed on medial and lateral malleoli, respectively
P1MT, P5MT, P3MT: Markers placed on the base of the 1^st^ and 5^th^ metatarsals, and between the base of the 3^rd^ and the 4^th^ metatarsals, respectively
D1MT, D5MT: Markers placed on the first and the fifth metatarsal heads, respectively
TOE: Marker placed on the mid-point of the distal heads of the 2^nd^ and 3^rd^ metatarsals
FF1, FF2: markers not used for the analysis
